# Perioperative neurocognitive disorders: A narrative review focusing on diagnosis, prevention, and treatment

**DOI:** 10.1111/cns.13873

**Published:** 2022-06-01

**Authors:** Hao Kong, Long‐Ming Xu, Dong‐Xin Wang

**Affiliations:** ^1^ Department of Anesthesiology and Critical Care Medicine Peking University First Hospital Beijing China; ^2^ Outcomes Research Consortium Cleveland Ohio USA

**Keywords:** delirium, neurocognitive disorders, perioperative period, postoperative cognitive complications

## Abstract

Perioperative neurocognitive disorders (NCDs) refer to neurocognitive abnormalities detected during the perioperative periods, including preexisting cognitive impairment, preoperative delirium, delirium occurring up to 7 days after surgery, delayed neurocognitive recovery, and postoperative NCD. The Diagnostic and Statistical Manual of Mental Disorders‐5th edition (DSM‐5) is the golden standard for diagnosing perioperative NCDs. Given the impracticality of using the DSM‐5 by non‐psychiatric practitioners, many diagnostic tools have been developed and validated for different clinical scenarios. The etiology of perioperative NCDs is multifactorial and includes predisposing and precipitating factors. Identifying these risk factors is conducive to preoperative risk stratification and perioperative risk reduction. Prevention for perioperative NCDs should include avoiding possible contributors and implementing nonpharmacologic and pharmacological interventions. The former generally includes avoiding benzodiazepines, anticholinergics, prolonged liquid fasting, deep anesthesia, cerebral oxygen desaturation, and intraoperative hypothermia. Nonpharmacologic measures include preoperative cognitive prehabilitation, comprehensive geriatric assessment, implementing fast‐track surgery, combined use of regional block, and sleep promotion. Pharmacological measures including dexmedetomidine, nonsteroidal anti‐inflammatory drugs, and acetaminophen are found to have beneficial effects. Nonpharmacological treatments are the first‐line measures for established perioperative NCDs. Pharmacological treatments are still limited to severely agitated or distressed patients.

## Graphical abstract

Perioperative neurocognitive disorders constitute a great challenge for older patients scheduled for surgery because their occurrence is associated with increased morbidity and mortality as well as enormous medical costs. Preoperative risk stratification and perioperative risk reduction should be adopted for perioperative NCDs prevention and treatment.

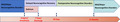

## 1 INTRODUCTION

Perioperative neurocognitive disorders (NCDs), especially postoperative delirium, delayed neurocognitive recovery, and postoperative NCD, are significant challenges to older patients scheduled for surgery.^1^ The resulting cognitive declines can persist for months or years and have a detrimental impact on self‐dependence, quality of life, risk of developing dementia, and even long‐term survival. With the aging population and growing number of surgeries, perioperative NCDs have become a public health problem and attracted worldwide attention.^2^ This review aims to discuss recent advances regarding perioperative NCDs, especially the diagnosis, prevention, and treatment.

## 2 DEFINITION AND CLASSIFICATION OF PERIOPERATIVE NCDS

**FIGURE 1:**
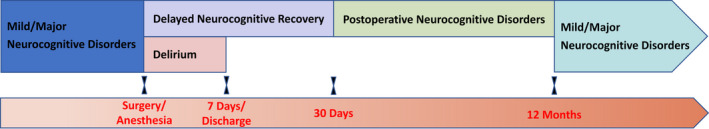
Classification of perioperative neurocognitive disorders

According to a recent consensus,^1^ “perioperative NCDs” are recommended to describe the overall situation identified during the pre‐ and postoperative periods. Perioperative NCDs are further classified into preexisting cognitive impairment or delirium, delirium occurred up to 7 days after surgery, cognitive decline diagnosed up to 30 days after surgery (delayed neurocognitive recovery), and cognitive decline diagnosed thereafter until 12 months (post‐operative NCD) (Figure 1).

It is suggested that cognitive screening should be performed in high‐risk patients before surgery.^3^ Those with preexisting impairment in one or more cognitive domains (complex attention, executive function, learning, memory, language, perceptual‐motor, and/or social cognition) are considered to have baseline NCD, which can be further classified as mild (mild cognitive impairment) or major disorder (dementia) according to the severity of impairment.

Delirium is a syndrome of acutely occurring and fluctuating changes in attention, level of consciousness, and cognitive function. It can occur preoperatively, but most often occurs within 7 days after surgery. Psychomotor disturbances (hyperactive, hypoactive, or mixed), perceptual disturbances (hallucination and delusion), emotional disturbances, and impaired sleep–wake cycle may also occur during delirium, although these features are not required for diagnosis.

Delayed neurocognitive recovery is a newly proposed term indicating a new‐onset cognitive decline within 30 days after surgery and replaces the traditional term “early postoperative cognitive dysfunction (POCD).” Because evidence shows that many and perhaps most patients recover completely from early postoperative cognitive impairment. Thus, the term “delayed neurocognitive recovery” interprets more accurately the meaning of potential recovery.

The term “postoperative NCD” now specifically refers to cognitive decline detected from 30 days after surgery to 12 months of follow‐up. After 12 months, the specifier “postoperative” is no longer attached when diagnosing cognitive decline, as the etiology cannot be reasonably attributed to the effects of prior anesthesia and surgery. Therefore, the term “NCD” is reused for the diagnosis after this timepoint.

The above diagnoses are made according to the Diagnostic and Statistical Manual of Mental Disorders fifth edition (DSM‐5).^1^ In most available studies, we note that POCD diagnosis was based on the presence of cognitive decline but not the DSM‐5 criteria. We, therefore, continue to use the term “POCD” rather than “postoperative NCD” in this paper when referring to previous results regarding cognitive decline occurred between 30 days to 12 months after surgery.

## 3 EPIDEMIOLOGY AND OUTCOMES OF PERIOPERATIVE NCDS

Numerous previous studies investigated the occurrence of postoperative delirium, delayed neurocognitive recovery, and POCD. The reported incidences vary widely due to the differences in patient populations and surgical types, the heterogeneity in the composition of test batteries, the criteria or definition for diagnoses, the timing of assessment, and the nature of studies (retrospective studies often underestimate the incidence when compared with prospective ones).

### 3.1 Postoperative delirium

In general adult patients, the incidence of postoperative delirium is 2.5%–4.5%.^4^, ^5^ In patients aged 60 years or above, the incidence of postoperative delirium is increased to 12.0%–23.8%.^6^, ^7^ The type and complexity of surgery also greatly affect the occurrence of delirium. According to the report from the American College of Surgeons National Surgical Quality Improvement Program, among the 20,212 older patients, the incidence of delirium was 13.7% after cardiothoracic surgery, 13.0% after orthopedic surgery, 13.0% after general surgery, 11.4% after vascular surgery, 8.0% after neurosurgery, 7.1% after plastics and otolaryngology, 6.6% after urology, and 4.7% after gynecology.^7^ Other studies also reported high incidence of delirium in patients following cardiovascular surgery (15.3–23.4%),^8^, ^9^, ^10^ hip fracture surgery (16.9%),^11^ and emergency surgery (22.7–26%),^12^, ^13^ and in those who were admitted to intensive care unit after surgery (24.4%).^14^

Delirium is an imperative predictor of adverse outcomes. In the early postoperative period, delirium is associated with a prolonged hospital stay, increased institutional discharge, and higher 30‐day readmission; the outcomes of delirium are additive to those of other major complications.^15^, ^16^ Mounting evidence suggests that delirium is associated with an increased risk of perioperative and long‐term mortality.^16^, ^17^, ^18^, ^19^ Among survivors, delirium is significantly linked to cognitive decline, both early (1 month after surgery) and long‐term after surgery^20^, ^21^; delirium is also associated with a decrease in health‐related quality of life^18^ and an increased risk of dementia.^16^, ^22^

### 3.2 Delayed neurocognitive recovery and POCD

An early multicenter study, the International Study of Post‐Operative Cognitive Dysfunction (ISPOCD1), investigated the occurrence of cognitive decline in 1218 elderly patients after major abdominal and orthopedic surgery. Delayed neurocognitive recovery was diagnosed in 25.8% of patients at 1 week after surgery; POCD was diagnosed in 9.9% of patients at 3 month after surgery.^23^ In another prospective study of 1064 adult patients following noncardiac surgery, the incidence of delayed neurocognitive recovery was 34.5% at hospital discharge, and that of POCD was 7.0% at 3‐month follow‐up.^24^ In a systematic review involving 24 studies with 8314 patients following noncardiac and non‐neurological surgeries, the pooled incidence of POCD at 3 months was 11.7%.^25^

The majority of patients who developed early cognitive decline recover with time, only about 1/6 to 1/2 of them have POCD during the follow‐up period.^23^, ^24^, ^26^, ^27^ However, delayed neurocognitive recovery is also associated with adverse outcomes. For example, patients with cognitive decline 1 week after surgery are more likely to leave the labor market and depend on social transfer payment.^28^ A recent study reported that those with delayed neurocognitive recovery between 3 and 5 days after surgery had more self‐reported cognitive impairment in memory, attention, action, and perception at 12 month after radical prostatectomy.^29^ It seems that patients with POCD have even worse outcomes. For example, studies found that patients with POCD at 3 months after surgery had twice the proportion of new impairment in activities of daily living when compared with those without^30^; they also had a higher risk of long‐term mortality.^24^, ^28^ Available data do not find a significant association between delayed neurocognitive recovery or POCD and long‐term dementia^31^; further studies are required to clarify this issue.

## 4 RISK FACTORS OF PERIOPERATIVE NCDS

The etiologies of perioperative NCDs are multifactorial^32^ and can be classified as predisposing and precipitating factors. The risk factors for delirium, delayed neurocognitive recovery, and POCD are significantly overlapped and are presented in Table 1.^9^, ^10^, ^14^, ^20^, ^23^, ^24^, ^25^, ^33^, ^34^, ^35^, ^36^, ^37^, ^38^, ^39^, ^40^, ^41^, ^42^, ^43^, ^44^, ^45^, ^46^, ^47^, ^48^, ^49^, ^50^, ^51^, ^52^, ^53^, ^54^, ^55^, ^56^, ^57^, ^58^, ^59^, ^60^, ^61^, ^62^, ^63^, ^64^, ^65^, ^66^, ^67^, ^68^, ^69^, ^70^, ^71^, ^72^, ^73^, ^74^, ^75^, ^76^, ^77^, ^78^, ^79^, ^80^, ^81^, ^82^, ^83^, ^84^, ^85^, ^86^, ^87^, ^88^, ^89^, ^90^ The development of perioperative NCDs results from the interaction of multiple factors. Many prediction models have been developed to predict the risk of perioperative NCDs.^41^, ^91^, ^92^, ^93^, ^94^, ^95^, ^96^ However, none of these models have been externally validated or widely accepted for clinical use.

**TABLE 1:** Risk factors of perioperative neurocognitive disorders

Risk factors	Delirium	Delayed neurocognitive recovery and POCD
Predisposing factors	Advanced age^9^, ^14^, ^33^, ^34^, ^35^, ^36^, ^37^, ^38^, ^39^, ^40^, ^41^, ^42^, ^43^	Advanced age^23^, ^24^, ^25^, ^38^, ^45^, ^68^, ^69^, ^70^, ^71^, ^72^
	Lower educational level^44^	Lower educational level^23^, ^24^, ^25^, ^61^, ^70^, ^73^
	Functional status: Cognitive impairment,^9^, ^10^, ^14^, ^35^, ^36^, ^39^, ^41^, ^43^, ^45^, ^46^ hearing impairment,^46^ frailty^38^, ^47^, ^48^, ^49^	Functional status: cognitive impairment,^69^, ^71^, ^74^, ^75^, ^76^ frailty^38^, ^77^
	Comorbidities: depression,^9^, ^35^, ^37^, ^39^, ^45^, ^50^ psychiatric illness,^36^, ^39^, ^40^ cerebrovascular disease,^10^, ^36^, ^37^, ^42^, ^45^, ^46^ parkinsonism,^42^ heart failure,^33^, ^39^ hypertension,^9^, ^39^, ^44^ mitral valve disease,^44^ diabetes,^14^, ^35^, ^37^ obstructive sleep apnea,^36^ pulmonary diseases,^37^, ^39^ kidney disease,^10^, ^39^ number of medications^37^, ^51^	Comorbidities: depression,^45^ cerebrovascular disease,^24^, ^78^ heart failure,^69^ hypertension,^69^, ^79^ diabetes,^69^, ^72^, ^80^, ^81^ renal failure^72^
	Comorbidity scores: higher ASA grade,^10^, ^34^, ^40^ NYHA functional class III or IV,^35^ higher EuroSCORE,^45^ higher Charlson Comorbidity Index^52^	Comorbidity scores: higher ASA grade,^82^ higher EuroSCORE^69^
	Alcohol abuse^34^, ^39^, ^40^	Alcohol abuse^79^, ^83^
	Nutritional status: malnutrition/low albumin,^14^, ^34^, ^37^, ^40^, ^41^, ^51^, ^53^ low preoperative hematocrit,^37^, ^42^, ^51^ vitamin D deficiency^54^, ^55^	Nutritional status: anemia,^70^ vitamin D deficiency^55^, ^84^
Precipitating factors	Preoperative preparation: long‐duration fluid fasting,^56^, ^57^ preoperative pain^39^, ^50^	
	Perioperative medications: anticholinergic drugs,^58^ benzodiazepines,^14^, ^59^ opioid use,^33^, ^37^, ^39^ use of pethidine^60^	Perioperative medications: anticholinergic drugs,^85^ opioid use^86^, ^87^
	Intraoperative factors/management: deep anesthesia,^61^ intraoperative blood loss/blood transfusion,^34^, ^36^, ^37^, ^40^, ^51^ cerebral oxygen desaturation,^62^, ^63^, ^64^ hypotension,^65^, ^66^ hypothermia^67^	Intraoperative factors/management: deep anesthesia,^61^, ^88^ intraoperative bleeding,^79^ cerebral oxygen desaturation^64^, ^89^, ^90^
	Surgical management: abdominal/orthopedic surgery or higher Surgical Apgar score,^41^, ^42^ long‐duration surgery^37^, ^51^	Surgical management: major surgery,^71^ long‐duration surgery^23^, ^69^, ^78^
	Postoperative management: severe pain,^40^ long‐duration mechanical ventilation,^14^, ^35^ prolonged stay in the intensive care unit^35^, ^45^	Postoperative management: severe pain,^79^ occurrence of delirium,^20^, ^25^, ^45^ prolonged stay in the intensive care unit^45^

Abbreviations: ASA, American Society of Anesthesiologist; EuroSCORE, European System for Cardiac Operative Risk Evaluation score; NYHA, New York Heart Association; POCD, postoperative neurocognitive disorder.

## 5 DIAGNOSIS OF PERIOPERATIVE NCDS

### 5.1 Diagnosis of delirium

According to the recent consensus, postoperative delirium is considered a particular category consistent with DSM‐5 terminology and occurs in the immediate postoperative period.^1^ In other words, postoperative delirium is a new‐onset event that occurs during a hospital stay for up to 7 days after surgery or until discharge and meets the diagnostic criteria of DSM‐5.^1^, ^97^ Delirium occurring after discharge is no longer specified as “postoperative” unless persistent from the in‐hospital postoperative period. Attention should be paid to differentiating emergence delirium from postoperative delirium when making the diagnosis. Emergence delirium occurs during or immediately after emergence from general anesthesia and usually resolves within minutes or hours, whereas postoperative delirium mainly occurs 24–72 h after surgery and usually resolves within hours to days.^98^ During research and clinical practice, emergence delirium is mainly monitored during the stay in the post‐anesthesia care unit, whereas postoperative delirium is mainly monitored from the first postoperative day.^67^, ^99^

DSM‐5 is the gold standard for diagnosing postoperative delirium. However, the use of DSM‐5 is impractical by non‐psychiatric practitioners. So far, more than 20 diagnostic tools have been introduced and validated to facilitate the diagnosis of delirium.^100^, ^101^, ^102^ Among these, the frequently used screening tools including the Confusion Assessment Method (CAM),^103^ the Confusion Assessment Method for the Intensive Care Unit (CAM‐ICU),^104^ the Brief Confusion Assessment Method (bCAM),^105^ the 3‐Minute Diagnostic Interview for Delirium using the Confusion Assessment Method (3D‐CAM),^106^ the Intensive Care Delirium Screening Checklist (ICDSC),^107^ and the 4A's Test (4AT).^108^ Several instruments have been validated to measure delirium severity. The Memory Delirium Assessment Scale (MDAS),^109^ the delirium Rating Scale‐Revised‐98 (DRS‐R‐98),^110^ and the Confusion Assessment Method‐Severity (CAM‐S)^111^ are the three most commonly used tools for assessing delirium severity. The characteristics of these tools are presented in Table 2.^97^, ^104^, ^105^, ^106^, ^107^, ^108^, ^109^, ^110^, ^111^, ^112^, ^113^, ^114^, ^115^

**TABLE 2:** Characteristics of the frequently used delirium screening tools

Screening tools	Based criteria	No. of items	Target patients	Time taken (min)	Sensitivity (%)	Specificity (%)	Interrater reliability
Diagnosis of delirium							
DSM‐5	—	5 criterion	General medical and surgical	30	100^97^	100^97^	*κ* = 0.73^112^
CAM	DSM‐3R	4 core features 9 items	General medical and surgical	5	43–94^113^	83–100^113^	*κ* = 0.88^114^
CAM‐ICU	CAM	4 core features	Critically ill, especially intubated and sedated	2	93–100^104^	98–100^104^	*κ* = 0.96^104^
bCAM	CAM	4 core features 7 items	Emergency	<2	78–84^105^	96–97^105^	*κ* = 0.88^105^
3D‐CAM	CAM	4 core features 20 items	General medical and surgical	3	95^106^	94^106^	95%^a^, ^106^
ICDSC	DSM‐4	8 items	Critically ill	<5	99^107^	64^107^	NR
4AT	DSM‐4	4 items	General medical and surgical	<2	90^108^	84^108^	NR
Severity of delirium							
MDAS	DSM‐4	10 items	General medical and surgical	10–15	Correlations among three tools^b^: MDAS and DRS‐R‐98, *r* = 0.91; MDAS and CAM‐S LF, *r* = 0.91; MDAS and CAM‐S SF, *r* = 0.87; DRS‐R‐98 and CAM‐S LF, *r* = 0.88; DRS‐R‐98 and CAM‐S SF, *r* = 0.82; CAM‐S LF and CAM‐SF, *r* = 0.96.^115^	ICC = 0.92^109^	
DRS‐R‐98	DSM‐3	16 items (3 items for diagnosis and 13 items for severity)	General medical and surgical	20–30		DRS‐R‐98 total, ICC = 0.98; DRS‐R‐98 severity, ICC = 0.99^110^	
CAM‐S	CAM	SF, 4 core features; LF, 10 core features	General medical and surgical	LF, 10; SF, 5		LF, ICC = 0.88; SF, ICC = 0.92^111^	

Abbreviation: 3D‐CAM, 3‐Minute Diagnostic Interview for Delirium using the Confusion Assessment Method; 4AT, The 4‐item Assessment Test; bCAM, Brief Confusion Assessment Method; CAM, Confusion Assessment Method; CAM‐ICU, Confusion Assessment Method for the Intensive Care Unit; CAM‐S, Confusion Assessment Method‐Severity; CI, confidential interval; DRS‐R‐98, Delirium Rating Scale, Revised Version; DSM, Diagnostic and Statistical Manual; ICC, intraclass correlation coefficient; ICDSC, Intensive Care Delirium Screening Checklist; LF, long‐form; MDAS, Memory Delirium Assessment Scale; NR, not reported; SF, short‐form.

^a^ Indicating interrater agreement.

^b^ There is no gold standard for assessing the severity of delirium.

**FIGURE 2:**
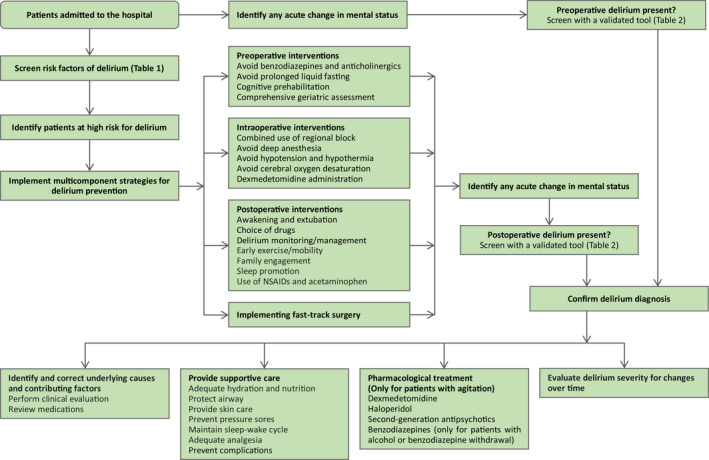
Suggested algorithm for assessment, prevention, and treatment of delirium

Perioperative delirium assessment is usually performed in high‐risk patients or in those who suffer any acute change in mental status (Figure 2).

### 5.2 Diagnosis of delayed neurocognitive recovery and postoperative NCD/POCD

The objective evidence for NCD is “modest (mild NCD) or significant (major NCD) cognitive decline from the previous level of performance in one or more cognitive domains (complex attention, executive function, learning, and memory, language, perceptual‐motor, or social cognition).”^97^ Traditionally, a battery of neurocognitive tests is used to evaluate the function of different cognitive domains. Among the individual neurocognitive tests, the digit span test (including forward and backward subtests), the trail‐making test part A, and the digit symbol substitution test are the most commonly used.^116^, ^117^ However, accomplishing a battery of tests is time‐consuming. To simplify the diagnosing process, several brief tools are being used to detect mild cognitive impairment; among them, the Montreal Cognitive Assessment (MoCA), the Addenbrooke's Cognitive Exam (ACE‐III), and the Quick MCI Screen (Qmci) are regarded as promising in the perioperative environment (Table 3).^118^, ^119^, ^120^, ^121^, ^122^, ^123^, ^124^, ^125^, ^126^, ^127^, ^128^, ^129^

**TABLE 3:** Characteristics of brief tools for detecting mild cognitive impairment

Screening tools	Domains included	Time taken (min)	Total score	Cut‐off score	Sensitivity (%)	Specificity (%)	Interrater reliability
MoCA	Visuospatial/executive, language, memory, and delayed recall	10–15 min	30	23	78–87^119^, ^120^, ^121^	73–98^119^, ^120^, ^121^	0.96^119^
				24	80–89^122^, ^123^, ^124^, ^125^	64–83^122^, ^123^, ^124^, ^125^	
				26	90–96^120^, ^124^, ^126^, ^127^	58–87^120^, ^124^, ^126^, ^127^	
ACE‐III	Visuospatial, verbal fluency, language, memory, and attention	16 min	100	88	75–80^125^, ^128^, ^129^	86–92^125^, ^128^, ^129^	0.996^128^
Qmci	Orientation, word registration, clock drawing, delayed recall, verbal fluency, and logical memory	3–5 min	100	52	69^127^	97^127^	1.0^127^
				62	90^124^	87^124^	
				65	94^124^	80^124^	

Abbreviation: MoCA, Montreal Cognitive Assessment; ACE, Addenbrooke's Cognitive Exam; Qmci, Quick Mild Cognitive Impairment Screen.

According to the DSM‐5, mild NCD requires a decline of 1 to 2 standard deviations (for test results with normal distribution) or 3rd to 16th percentile (for test results with nonnormal distribution); major NCD requires a decline of >2 standard deviations (normal distribution) or 3rd percentile or below (nonnormal distribution).^1^, ^97^ Normal or baseline values are required when using the above cut‐points to quantify the extent of cognitive changes. The z‐score calculation is widely accepted when both baseline and control groups are available.^130^ For each test, the mean practice effect is subtracted from the difference between preoperative and postoperative test scores; the result is then divided by the control group standard deviation to obtain a *z*‐score. The *z*‐scores of all tests in an individual patient are summarized and divided by the standard deviations for this sum of *z*‐scores in the control subjects, creating a composite z‐score. Cognitive decline is defined when two *z*‐scores in individual tests or the composite z‐score are −1.96 or less.^130^

The diagnosis of POCD is confined to research and requires an only objective decline in cognition.^116^ Different from POCD, the diagnosis of NCD requires the presence of cognitive concerns and the evaluation of daily activity in addition to the evidence of cognitive decline.^97^ Cognitive concerns can be provided by individuals, informants, or clinicians. History‐taking should be performed to assess cognitive concerns before and after surgery. Daily activity can be assessed with an appropriate tool for subtle changes; it can also be self‐reported or informant‐reported. Mild NCD requires that the daily function is overall maintained, whereas major NCD requires a decline of daily function.^1^ Because of pharmacological and surgical interventions, assessing cognitive symptoms and daily activity is problematic during a hospital stay and even after hospital discharge until a full recovery is achieved. Therefore, the Nomenclature Consensus Working Group^1^ recommends that the term “delayed neurocognitive recovery” is used to describe the state of “NCD” for up to 30 days after surgery and that the term “postoperative mild/major NCD” is used thereafter until 12 months after surgery. The specifier “postoperative” is no longer used beyond 12 months unless the diagnosis is made within 12 months.

Cognitive function assessment is usually performed in high‐risk patients during preoperative and postoperative periods (Figure 2).

## 6 PREVENTION FOR PERIOPERATIVE NCDS

**FIGURE 3:**
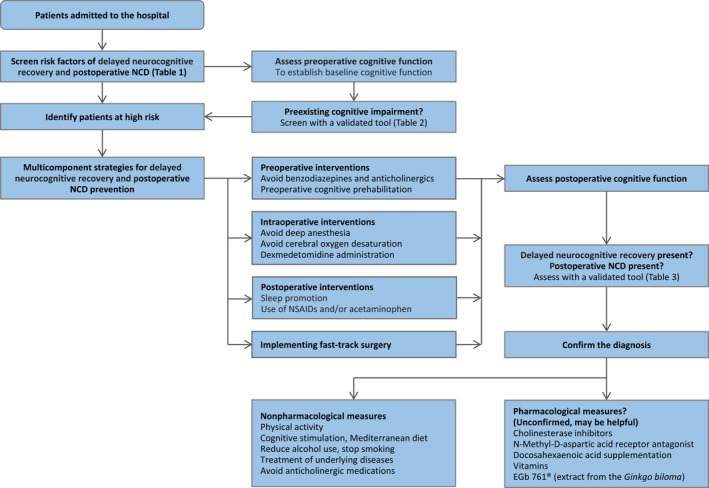
Suggested algorithm for assessment, prevention, and treatment of delayed neurocognitive recovery and postoperative neurocognitive disorders

As expected, multiple strategies implemented by a multidisciplinary team have yielded substantial benefits. Some single interventions have demonstrated efficacies (Figures 2 and 3).

### 6.1 Prevention for postoperative delirium

#### 6.1.1 Preoperative measures

##### 6.1.1.1 Avoiding benzodiazepines and anticholinergics

Benzodiazepines produce amnesia, anxiolysis, and sedation and are commonly used to treat anxiety and insomnia. Benzodiazepines also play a vital role in anesthetic practice. However, perioperative exposure to benzodiazepines is associated with delirium.^131^, ^132^, ^133^, ^134^ Guidelines recommend avoiding routine premedication with benzodiazepines, except for patients with severe anxiety, to reduce the risk of postoperative delirium.^135^, ^136^ Dexmedetomidine may be a suitable substitute for benzodiazepines but requires further investigation.^137^

Anticholinergics have long been identified as an important risk factor for postoperative delirium and should be avoided.^58^, ^138^

##### 6.1.1.2 Avoiding prolonged liquid fasting

Prolonged liquid‐fasting results in discomfort feelings such as thirst and anxiety and increases the incidence of postoperative nausea and vomiting. Long duration of preoperative liquid‐fasting is also associated with an increased risk of emergence delirium in both adult^56^ and pediatric patients.^57^ Shortening the duration of liquid‐fasting to 2 h, as recommended by guidelines,^139^ may reduce the incidence of emergence delirium but requires further validation.

##### 6.1.1.3 Cognitive prehabilitation

Low cognitive reserve, as manifested by low educational levels, less participation in cognitive activities (such as reading, writing, playing games, emailing, and singing), or low cognitive test scores, is a crucial risk factor for developing delirium after surgery.^140^, ^141^ Cognitive training effectively maintains cognitive function in healthy older people and improves cognitive function in patients with dementia.^142^, ^143^ In a recent trial, 251 older patients undergoing major noncardiac surgery were randomly assigned to receive no cognitive training or 1‐h cognitive exercise for 10 consecutive days before surgery; the results showed that cognitive prehabilitation reduced the incidence of postoperative delirium from 23.0% to 14.4%, although the differences did not reach statistical significance (*p* = 0.08).^144^ More extensive trials are warranted to verify the effects.

##### 6.1.1.4 Comprehensive geriatric assessment

Comprehensive geriatric assessment is a coordinated multidisciplinary assessment of the medical, psychosocial, environmental, and functional concerns in risky older patients. The purpose is to identify risk factors and establish a treatment plan. A recent comment suggested that a combined preoperative assessment for cognition, frailty, and mental disorders should be performed for older patients to identify those at high risk of postoperative neurological complications.^3^ Available studies showed that comprehensive geriatric assessment reduces delirium and improves outcomes in patients with hip fracture,^145^, ^146^ possibly by the multicomponent intervention.^147^

#### 6.1.2 Intraoperative measures

##### 6.1.2.1 Neuraxial anesthesia and peripheral nerve block

Neuraxial anesthesia has the advantages of reducing opioid consumption, blunting stress response, and providing better analgesia when compared with general anesthesia alone. Neuraxial anesthesia is recommended over general anesthesia for patients undergoing major surgery to improve postoperative outcomes.^148^, ^149^

Lower limb orthopedic surgery can be performed with either neuraxial anesthesia or general anesthesia. Early studies regarding the effect of neuraxial anesthesia in hip fracture patients did not reach a conclusion.^150^ A population‐based cohort study found that the use of neuraxial anesthesia was associated with less delirium following hip/knee arthroplasties.^5^ However, two recent large sample size trials reported neutral results, that is, neuraxial anesthesia compared with general anesthesia did not reduce delirium in patients following hip fracture surgery.^151^, ^152^ So neuraxial anesthesia is not superior to general anesthesia regarding postoperative delirium in this patient population.

Most major surgeries are performed under general anesthesia. A 2014 meta‐analysis showed that compared with general anesthesia alone, those given combined epidural‐general anesthesia developed less cardiopulmonary and gastrointestinal complications and had lower mortality after surgery.^148^ In a recent randomized trial of 1802 patients following major thoracic and abdominal surgeries, those given combined epidural‐general anesthesia had one‐third as much delirium when compared with those given general anesthesia alone.^153^ Combined epidural‐general anesthesia should be considered in patients undergoing major surgery and at high risk of postoperative delirium.

Peripheral nerve block is increasingly used as an alternative to neuraxial anesthesia; when compared with neuraxial block, it provides a slightly less analgesic effect but has fewer side effects, including hypotension and urinary retention.^154^, ^155^, ^156^ Studies reported that femoral nerve block reduced the incidence of postoperative delirium in patients following hip fracture surgery^157^ or total knee replacement.^158^ A recent meta‐analysis of 8 randomized controlled trials showed that regional nerve block reduced perioperative delirium in older hip fracture patients, but the effect was limited to those without preoperative cognitive impairment.^159^ The effect of peripheral nerve block on postoperative delirium requires further validation.

##### 6.1.2.2 Choice of anesthetics during general anesthesia

Both intravenous and inhalational anesthetics are commonly used during general anesthesia. A recent meta‐analysis included 12 high‐quality randomized trials with 1440 patients; the results showed that propofol‐based intravenous maintenance compared with inhalational maintenance reduced emergence delirium by a half.^160^ Emergence delirium is found to be associated with postoperative delirium.^67^ However, the impact of anesthetic choice on postoperative delirium remains unclear. In a 2018 meta‐analysis, only five trials with 321 patients compared the effect of anesthetic maintenance agents on postoperative delirium and found no significant difference.^161^ A 2019 meta‐analysis included trials in patients undergoing coronary artery bypass grafting; no difference was found in postoperative delirium between volatile versus intravenous anesthesia.^162^

Xenon is a noble gas with anesthetic properties, mainly by inhibiting N‐methyl‐D aspartate receptors in the central nervous system. Xenon anesthesia provides more stable intraoperative hemodynamics and faster emergence from anesthesia than propofol and other volatile agents.^163^ In an early pilot trial, patients given xenon anesthesia had less delirium following cardiac surgery.^164^ However, a subsequent trial did not find a difference between the xenon and sevoflurane groups.^165^ In a phase II randomized trial of 256 elderly patients undergoing hip fracture surgery, xenon anesthesia did not reduce postoperative delirium.^166^

Therefore, the impact of anesthetic maintenance agents on postoperative delirium deserves further study.

##### 6.1.2.3 Monitoring of anesthesia depth

Electroencephalogram‐based anesthetic depth, such as bispectral index (BIS), is increasingly monitored during general anesthesia. Studies suggest that BIS‐guided anesthesia maintenance avoids unnecessary deep anesthesia, decreases anesthetic consumption, and improves emergence from anesthesia after surgery.^167^, ^168^, ^169^ Meta‐analyses showed that BIS‐guided anesthesia compared with no BIS‐guided anesthesia decreased delirium at 1 day after surgery.^170^, ^171^ Another meta‐analysis of 10 randomized controlled trials also showed that light anesthesia was associated with a decrease in postoperative delirium when compared with deep anesthesia.^61^ A recent trial reported similar results, that is, patients given light anesthesia developed less postoperative delirium.^172^ Thus, anesthesia depth should be monitored to avoid deep anesthesia in high‐risk patients.

##### 6.1.2.4 Avoiding intraoperative hypotension and hypothermia

Accumulating evidence shows that even short durations of intraoperative hypotension are associated with adverse outcomes, including acute kidney injury, myocardial injury, and death.^173^, ^174^ Some retrospective cohort studies reported an association between intraoperative hypotension and postoperative delirium.^66^, ^175^ However, a meta‐analysis of randomized trials did not find a significant association between intraoperative hypotension and the risk of postoperative delirium.^176^ Two systematic reviews also conclude that there is no convincing evidence that intraoperative hypotension is a risk factor of postoperative delirium.^177^, ^178^ Nevertheless, intraoperative hypotension should be avoided considering the harmful effects on other organs.

Hypothermia is common during general anesthesia and surgery.^179^ Intraoperative hypothermia is associated with adverse outcomes such as increased bleeding, more wound infection, and increased cardiac events.^180^ Furthermore, intraoperative hypothermia prolongs the duration of anesthetic agents and delays postanesthesia recovery.^179^, ^181^ In a prospective cohort study, intraoperative hypothermia (<36°C) was an independent risk factor of the emergence of delirium.^67^ A recent retrospective study found a significant relationship between perioperative hypothermia and postoperative delirium and a complex relationship among perioperative hypothermia, age, ASA class, and postoperative delirium.^182^ Avoiding intraoperative hypothermia may help to reduce postoperative delirium but requires further validation.

##### 6.1.2.5 Monitoring of cerebral oxygen saturation

In critically ill patients, dysfunction of cerebral autoregulation is associated with delirium.^183^ Indeed, cerebral hypoxia is found to be a potential risk factor for perioperative NCDs. For example, studies reported that patients with low preoperative cerebral oxygen saturation had a higher incidence of postoperative delirium^184^, ^185^; intraoperative cerebral oxygen desaturation was associated with increased postoperative delirium.^62^, ^63^, ^64^ A pairwise and network meta‐analysis included 12 randomized trials with 1626 patients investigating the effect of brain near‐infrared spectroscopy‐based intervention; the results showed that cerebral oxygenation‐guided management reduced delirium after cardiac surgery but not major noncardiac surgery.^186^ Cerebral oxygenation‐guided management may be helpful in patients undergoing cardiac surgery. The optimal threshold and the effect in noncardiac surgery patients require further evaluation.

##### 6.1.2.6 Dexmedetomidine

Dexmedetomidine is a highly selective α2‐receptor agonist with anxiolytic, sedative, and analgesic properties. It has been widely used as an adjuvant during anesthesia and for sedation in the ICU. A 2021 meta‐analysis of 13 randomized trials showed that perioperative administration of dexmedetomidine, either intraoperatively or intra‐ and postoperatively, significantly reduced delirium in elderly patients following noncardiac surgery.^187^ Studies in cardiac patients remains controversial. A recent meta‐analysis included 30 randomized trials comprising 4090 patients undergoing cardiac surgery. With unselected trials, dexmedetomidine compared with control decreased the incidence of postoperative delirium (RR 0.62, 95% CI 0.44–0.86, *p* = 0.005); however, the difference was not statistically significant after excluding trials at high risk of bias (RR 0.71 95% CI 0.49–1.03, *p* = 0.070).^188^

#### 6.1.3 Postoperative measures

##### 6.1.3.1 Nonpharmacologic strategies

Nonpharmacologic strategies targeting multiple risk factors are the first‐line measures for preventing postoperative delirium.^189^ The relevant interventions usually include reorientation and cognitive stimulation, sleep enhancement, early mobility and exercise, vision and hearing optimization, family engagement and empowerment, and early oral intake or nutrition. A recent meta‐analysis shows that multicomponent nonpharmacological interventions effectively reduce the incidence of delirium; the interventions may also shorten the length of hospital stay.^190^ The Awakening and Breathing Coordination, Choice of drugs, Delirium monitoring and management, Early mobility, and Family engagement (ABCDEF) bundle are designed for critically ill patients in the ICU; it effectively reduces delirium and promotes weaning from mechanical ventilation.^191^, ^192^ A subsequent large sample size study also showed that implementation of the bundle is associated with meaningful improvement in the outcomes, including delirium and survival.^193^

##### 6.1.3.2 Sleep promotion

Sleep disturbances frequently occur in patients after surgery. Poor sleep quality is associated with an increased risk of delirium.^194^ Sleep promotion is an essential component of delirium prevention strategies.^195^ As mentioned above, nonpharmacological interventions are first‐line measures for sleep promotion; these include maintaining a quiet and dim environment, decreasing interruptions from care activities at night, and using overnight eye masks and earplugs. Evidence shows that the use of earplugs and/or eye masks may be beneficial to improve sleep and reduce delirium in ICU patients.^196^

As part of a multimodal approach, pharmacotherapy may be necessary to improve sleep. Nonbenzodiazepine agents are preferred for this purpose.^197^ Melatonin and melatonin receptor agonists (ramelteon) are now used to consolidate the circadian rhythm. A 2021 meta‐analysis of 14 trials with 1712 participants reported that the use of melatonin or ramelteon is associated with improved sleep quality and reduced delirium in surgical and ICU patients.^198^ However, a recent multicenter trial of 847 patients (including 1/4 surgical patients) showed that enteral melatonin initiated within 48 h of ICU admission did not reduce delirium.^199^ Suvorexant is a new orexin receptor antagonist approved for the treatment of insomnia. It has been used to improve sleep in hospitalized patients. A systematic review of 6 studies with 645 acutely hospitalized patients (including surgical patients) showed that those given suvorexant for sleep promotion developed less delirium and had a shorter length of hospital stay.^200^

Dexmedetomidine exerts sedative effects by activating the endogenous sleep‐promoting pathway^201^ and is thus used to promote sleep. In critically ill patients with mechanical ventilation, night‐time infusion of sedative‐dose dexmedetomidine improves sleep by increasing sleep efficacy and stage 2 sleep and maintaining circadian rhythm.^202^ Night‐time low‐dose dexmedetomidine infusion in ICU patients without mechanical ventilation also improves sleep architecture and subjective sleep quality.^203^ In line with these, night‐time dexmedetomidine reduced delirium in ICU patients.^99^, ^204^ It is worthy to note that an even lower dose of dexmedetomidine, such as used as an adjuvant in the patient‐controlled analgesia pump, also improves sleep quality after surgery.^205^ Dexmedetomidine supplemented analgesia may also reduce delirium but requires further investigation.^206^

##### 6.1.3.3 Nonsteroidal anti‐inflammatory drugs and acetaminophen

Severe pain is associated with an increased risk of delirium. Inflammation provoked by surgery plays a vital role in the pathogenesis of postoperative delirium. Nonsteroidal anti‐inflammatory drugs (NSAIDs) and acetaminophen are commonly used adjuvant analgesics; NSAIDs also effectively alleviate the degree of surgery‐related inflammatory response. These drugs are therefore investigated to prevent postoperative delirium. In a randomized trial of elderly patients undergoing hip or knee arthroplasty, parecoxib supplemented morphine analgesia almost halves the incidence of postoperative delirium.^207^ In older patients following cardiac surgery, intravenous acetaminophen reduced delirium by 64%.^208^ In a small sample size trial, flurbiprofen axetile supplemented analgesia decreased delirium in a subgroup of patients over 70 years after major noncardiac surgery.^209^ Thus, NSAIDs and acetaminophen should be considered for delirium prevention in patients without contraindications.

#### 6.1.4 Implementing fast‐track surgery

Fast‐track surgery (or enhanced recovery after surgery) is a multimodal approach to improve patient care by adopting a combination of evidence‐based interventions by a multidisciplinary team; the purpose is to expedite recovery after surgery. Strategies are adopted to reduce the surgical stress response and inflammation, facilitate physical rehabilitation, and enhance early return to normal circadian rhythm, all of which may improve postoperative cognitive functions. Studies reported that fast‐track surgery is associated with a reduced incidence of delirium in patients following hip and knee arthroplasty^210^, ^211^ and colorectal surgery.^212^ In a meta‐analysis, implementing enhanced recovery after surgery programs in hip fracture patients halved the incidence of postoperative delirium.^213^ Strategies for fast‐track surgery and enhanced recovery after surgery are recommended for postoperative delirium prevention.^135^

#### 6.1.5 Delirium prevention in patients with COVID‐19

Neurological manifestations may occur in COVID‐19 patients.^214^, ^215^ Encephalopathy, which is defined as global cerebral disturbance and expressed as either subsyndromal delirium, delirium, or coma, predicts adverse outcomes in patients with COVID‐19 infection.^216^ Delirium is common but often ignored in ICU patients with COVID‐19.^217^, ^218^ Identified risk factors include mechanical ventilation, use of restraints, infusion of benzodiazepines, opioids and vasopressors, and administration of antipsychotics; whereas family visitation (in person or online) was associated with less delirium.^218^ However, implementation of the ABCDEF bundle is challenging in this patient population due to multiple reasons, such as changes in critical care hierarchy and priorities, shortages of medical staff and personal protective equipment, reduced bedside presence, and increased use of deep sedation and neuromuscular blockade.^219^ Strategies to optimize bundle performance have been proposed.^219^, ^220^ According to recent study results, reducing benzodiazepine sedation and increasing family visit should be especially encouraged for delirium prevention in this patient population.^218^

### 6.2 Prevention for delayed neurocognitive recovery and postoperative NCD/POCD

#### 6.2.1 Preoperative measures

##### 6.2.1.1 Avoiding benzodiazepines and anticholinergics

Studies in the geriatric population showed that the use of benzodiazepine is associated with cognitive decline, dementia and Alzheimer's disease, although mixed findings exist; it seems that longer‐acting drugs, longer‐duration use, and early exposures are more harmful in this aspect.^221^ A randomized placebo‐controlled trial in children showed that premedication with low‐dose midazolam provoked significant short‐term impairment of cognitive function and amnesia enduring for 48 h.^222^ In a randomized trial of old patients, intraoperative sedation with midazolam increased delayed neurocognitive recovery at 7 days; but no difference in cognition function was found at 1 year.^223^ Routine premedication with benzodiazepine is not recommended in high‐risk patients.

Anticholinergics are also associated with an increased risk of cognitive decline after surgery and should be avoided in older patients.^224^, ^225^

##### 6.2.1.2 Cognitive prehabilitation

Preoperative cognitive training may augment cognitive reserve and improve cognitive recovery after surgery. In a randomized trial of 141 older patients undergoing gastrointestinal surgery, cognitive training for three 1‐h sessions before surgery reduced the incidence of delayed neurocognitive recovery from 36.1% to 15.9% (*p* < 0.05).^226^ The feasibility of perioperative cognitive training via a mobile device was also demonstrated in older adults undergoing cardiac surgery; those who received training reported improved memory and thinking ability.^227^ Other trials are under way to further examine the effect of cognitive prehabilitation on postoperative cognition.^228^

#### 6.2.2 Intraoperative measures

##### 6.2.2.1 Neuraxial anesthesia and peripheral nerve block

A small sample size trial compared general versus spinal anesthesia in hip fracture patients^229^; another small sample size trial compared general versus combined epidural‐general anesthesia in older patients undergoing abdominal surgery.^230^ Both reported no significant difference in delayed neurocognitive recovery after surgery.^229^, ^230^ As high‐quality studies are still lacking, conclusions cannot be reached regarding the effect of neuraxial anesthesia on postoperative cognition at the moment.

In a 2021 meta‐analysis of 122 studies on patients undergoing total hip/knee arthroplasty surgery, the use of peripheral nerve block (compared with no use) was associated with lower risks of numerous complications including cognitive dysfunction after surgery.^231^ In a small sample trial of patients following esophageal cancer surgery, the combined use of intercostal nerve block was associated with improved cognitive function recovery.^232^ Therefore, peripheral nerve block should be considered for patients without contraindications, either alone or in combination with neuraxial or general anesthesia.^233^ Further studies are required to confirm these findings.

##### 6.2.2.2 Choice of anesthetics during general anesthesia

In patients undergoing cardiac surgery, two small sample size trials reported that inhalational anesthesia was associated with less delayed neurocognitive recovery than propofol.^234^, ^235^ However, evidence is limited in this aspect. Subsequent meta‐analysis and a large sample size trial showed that anesthetic (volatile or total intravenous) choice does not change clinical outcomes including 1‐year mortality in cardiac surgery patients.^162^, ^236^

In patients undergoing non‐cardiac surgery, a 2018 meta‐analysis showed that propofol‐based total intravenous anesthesia may reduce delayed neurocognitive recovery with low‐certainty evidence^161^; whereas a 2019 meta‐analysis showed that propofol has more adverse effect on postoperative cognitive function in lung cancer patients.^237^ Subsequent trials also gave conflicting results, one reported less delayed neurocognitive recovery with intravenous anesthesia^130^; two others did not find significant differences.^238^, ^239^ Thus, available evidence does not suggest which anesthetic (propofol or volatile anesthetics) is better regarding postoperative cognitive recovery.

In a small sample size trial, Bronco et al^240^ reported that xenon anesthesia was associated with faster emergence and better cognitive recovery at 30 and 60 min after extubation. However, two other trials did not find difference in postoperative cognitive function at 6–12 and 66–72 h after surgery when comparing xenon versus sevoflurane^241^ or desflurane anesthesia.^242^ Current evidence does not support that xenon is superior to other anesthetics regarding postoperative cognitive function.

##### 6.2.2.3 Monitoring of anesthesia depth

A 2020 meta‐analysis including 8 trials showed that BIS‐guided (or BIS ≥50) anesthesia compared with no BIS‐guided (or BIS <50) anesthesia decreased POCD at 12 weeks after surgery.^171^ Two subsequent large sample size trials reported different results. A trial including 1232 older patients found that BIS‐guided anesthesia compared with usual care did not reduce cognitive impairment at 30 days.^243^ Another multicenter trial including 655 patients reported that patients given light anesthesia (BIS 50) had better cognitive function than those given deep anesthesia (BIS 35) at 1‐year follow‐up.^172^

There are also contrary results. For example, two small sample size trials comparing the effect of deep (BIS 30–45) versus light (BIS 45–65) total intravenous anesthesia reported that patients given deep anesthesia developed less delayed neurocognitive recovery.^244^, ^245^ The underlying reasons leading to the conflicting results are unclear but may include the difference in anesthetics. We note that most of the available studies were conducted in patients given inhalational or combined inhalational‐intravenous anesthesia; avoiding deep anesthesia may be helpful to improve postoperative cognitive recovery. However, the optimal anesthesia depth during total intravenous anesthesia requires further study.

##### 6.2.2.4 Avoiding intraoperative hypotension

A 2020 comprehensive review included 13 studies with 3017 patients undergoing noncardiac surgery,^177^ a 2021 systematic review included 6 studies with 1222 patients undergoing cardiac surgery^178^; both reviews did not find a significant association between intraoperative hypotension and postoperative cognitive impairment. Two other systematic review/meta‐analysis included studies of patients undergoing either cardiac or noncardiac surgeries also gave similar results.^176^, ^246^ Thus, available evidence did not find a conclusive association between intraoperative hypotension and postoperative cognitive decline.

##### 6.2.2.5 Monitoring of cerebral oxygen saturation

Accumulating evidence suggests that intraoperative cerebral oxygen desaturation is associated with an increased risk of delayed neurocognitive recovery.^27^, ^64^, ^89^, ^90^ In a 2018 meta‐analysis of 15 randomized trials comprising 1822 adult patients, management according to perioperative cerebral oxygenation monitoring reduced the occurrence of delayed neurocognitive recovery; however, the quality of evidence was low.^247^ In a recent meta‐analysis of 12 randomized trials with 1868 cardiac surgical patients, cerebral oxygenation‐guided intervention is associated with less delayed neurocognitive recovery.^248^ Monitoring of cerebral oxygen saturation may be especially helpful for the management of patients with dysfunctional cerebral autoregulation.^183^ A recent consensus recommends that cerebral oxygenation should be monitored in patients at risk of acute cerebral hypoperfusion.^249^

##### 6.2.2.6 Dexmedetomidine

Perioperative dexmedetomidine administration exerts beneficial effects on postoperative cognitive recovery. A meta‐analysis of 26 randomized trials confirmed that patients given perioperative dexmedetomidine had reduced incidence of delayed neurocognitive recovery within 7 days after surgery; the effect was partially attributed to the blunted inflammatory response.^250^ In a long‐term follow‐up study of 700 older patients admitted to ICU after surgery, those given dexmedetomidine had better cognitive function at 3 years.^251^ The effects of perioperative dexmedetomidine on early and long‐term neurocognitive outcomes deserve further investigation.

#### 6.2.3 Postoperative measures

##### 6.2.3.1 Nonpharmacological measures

In a recent randomized trial of 609 older patients scheduled for cardiovascular surgery, nonpharmacological measures for delirium prevention including reorientation, sleep aids and early mobilization did not reduce delayed neurocognitive recovery at 1 week and POCD at three and 12 months after surgery.^252^

##### 6.2.3.2 Sleep promotion

Sleep disturbance is associated with an increased risk of cognitive impairment after surgery, especially in older patients.^253^, ^254^ The effect of pharmacological sleep promotion on postoperative cognitive function remains insufficiently investigated. In a small sample trial with 54 patients following breast cancer surgery, use of melatonin for sleep promotion increased sleep efficiency and total sleep time but did not affect cognitive function.^255^ In a randomized trial of 139 older patients undergoing hip arthroplasty, supplemental melatonin from 1 day before until 5 days after surgery improved subjective sleep quality and cognitive function after surgery.^256^ Non‐sedative dose dexmedetomidine seems also effective for this purpose.^99^, ^203^ Three‐year follow‐up of a randomized trial found that low‐dose dexmedetomidine improved long‐term cognitive function and quality of life.^251^ Sleep promotion may be effective to improve postoperative cognitive recovery but deserves further validation.

##### 6.2.3.3 Nonsteroidal anti‐inflammatory drugs

Peripheral inflammation provoked by anesthetic exposure and surgery can induce blood–brain barrier disruption^257^; the resulting neuroinflammation is found to be an important underlying mechanism of perioperative NCDs.^258^ Therefore, intervention targeting perioperative inflammation may be effective in the prevention.^259^ A 2020 meta‐analysis included 8 randomized trials with 1106 patients preparing for orthopedic surgery; the results showed that perioperative parecoxib administration decreased the incidence of delayed neurocognitive recovery and improved Mini‐Mental Examination score early after surgery.^260^ Parecoxib should be considered in high‐risk patients without contraindications. Further studies are required to validate the effect of other nonsteroidal anti‐inflammatory drugs.

#### 6.2.4 Implementing fast‐track surgery

Implementing strategies of fast‐track surgery (or enhanced recovery after surgery) are associated with improved outcomes including reduced complications and shortened length‐of‐stay.^261^, ^262^ Observational studies showed that delayed neurocognitive recovery and POCD were rare after fast‐track hip‐ and knee‐ arthroplasty.^86^, ^263^ Whether a fast‐track surgery strategy can improve postoperative cognitive function needs to be verified by well‐designed randomized controlled trials.

## 7 TREATMENT FOR PERIOPERATIVE NCDS

### 7.1 Treatment for postoperative delirium

Diagnosed delirium can be classified into hypoactive, hyperactive, and mixed subtypes. No matter what subtype is, the basic treatment measures include correcting the underlying causes, providing supportive care, and controlling delirious symptoms.^100^, ^101^, ^264^ The underlying causes are usually multiple and should be identified and promptly addressed. The purposes of supportive care are to meet patients' daily needs and prevent complications. Multicomponent nonpharmacological measures for delirium prevention are also suggested for treatment purposes,^265^ but the effectiveness has not been verified.^266^, ^267^

Pharmacological treatment is only reserved for patients with severe agitation, that is, when delirium symptoms may threaten patients' safety or the safety of others.^101^, ^264^ Antipsychotics including haloperidol and second‐generation antipsychotics are traditionally used to control symptoms. However, recent meta‐analyses do not find differences in delirium duration, delirium severity, and other clinical outcomes when comparing antipsychotics versus placebo.^268^, ^269^, ^270^ The potential adverse effects of antipsychotics also cause concerns. For example, in a randomized trial investigating the treatment effect of antipsychotics in palliative care settings, patients given risperidone or haloperidol had higher delirium symptom scores and more extrapyramidal effects; patients given haloperidol also had worse overall survival.^271^ A meta‐analysis reported that antipsychotic therapy in dementia and general mental health care is associated with increased mortality.^272^ Therefore, antipsychotics should not be routinely used for delirium treatment.^270^

Studies regarding the treatment effect of dexmedetomidine for delirium are relatively rare. In a 2019 meta‐analysis of randomized trials investigating the effects of pharmacological treatment of delirium in critically ill adults, dexmedetomidine is the only drug that shortens delirium duration; it also shortens the duration of mechanical ventilation.^273^ However, the results were based on a single study.^273^ A 2021 meta‐analysis of 10 randomized trials and 5 observational studies evaluated the efficacy of dexmedetomidine in treating delirium; in one trial, dexmedetomidine shortened the duration of delirium; in 6 studies, it was associated with a lower point‐prevalence of delirium and a shorter time to resolution of delirium; in 4 trials, it was superior to haloperidol in shortening the time to delirium resolution.^274^ Dexmedetomidine is a promising agent for delirium treatment. On the contrary, benzodiazepines should not be used to treat agitated delirious patients unless their use is specifically indicated such as for the treatment of alcohol or benzodiazepine withdrawal.^275^

Treatment for postoperative delirium is summarized in Figure 2.

### 7.2 Treatment for delayed neurocognitive recovery and postoperative NCD

For established delayed neurocognitive recovery, many and perhaps most patients will recover completely from early postoperative cognitive impairment; only a fraction of patients evolve into postoperative NCD. It is necessary to inform patients of the occurrence of this event and track the changes in cognitive function.

There are no specific treatments for delayed neurocognitive recovery or postoperative NCD. Measures for cognitive decline, including mild cognitive impairment or dementia in the general population, are proposed for treatment purposes.^276^ Among these, nonpharmacological measures mainly target modifiable risk factors and include physical activity,^277^, ^278^ cognitive stimulation,^279^, ^280^ Mediterranean diet,^281^ reducing alcohol use,^282^ stop smoking,^283^ treatment of underlying diseases,^284^ and avoiding anticholinergic medications.^285^

Several pharmacological agents are approved for the treatment of Alzheimer's Disease and dementia with modest benefits, including cholinesterase inhibitors (donepezil, galantamine, and rivastigmine) and an N‐Methyl‐D‐aspartic acid receptor antagonist (memantine).^286^, ^287^ Currently, there are no pharmacological therapies approved to treat mild cognitive impairment. Donepezil does not show benefit in this patient population.^288^ Vitamins and docosahexaenoic acid supplementation may be helpful but requires further confirmation.^289^, ^290^ Multidomain intervention may be more beneficial.^291^ Many studies are still ongoing. EGb 761®, a special extract from the *Ginkgo biloba*, is suggested in the treatment of mild cognitive impairment and dementia.^292^, ^293^ However, more high‐quality evidences are needed to support the efficacy. It should be noted that available studies are rarely performed in perioperative patients; therefore, the effects of these interventions on postoperative cognitive recovery remain to be determined.

Potential treatment for delayed neurocognitive recovery and postoperative NCD is summarized in Figure 3.

## 8 SUMMARY

Perioperative NCDs are common and distressing conditions in older patients following surgery and are associated with increased morbidity and mortality and enormous medical costs. Advances in diagnosis have improved assessment and risk stratification. Identifying high‐risk patients and avoiding precipitating factors during the perioperative period is pivotal for prevention. Effective measures are emerging but require further validation. Ongoing efforts are needed on preventive and treatment strategies to optimize patients' outcomes.

## 9 AUTHORS’ CONTRIBUTIONS

HK participated in literature search, figure drawing, and initial writing and revision of the manuscript; L‐MX participated in literature search, figure drawing, and initial writing of the manuscript; D‐XW participated in literature search, critical revision, and approval of the final version. We confirmed that the manuscript had been read and approved by all named authors.

## 11 CONFLICT OF INTEREST

The authors declare no conflict of interest.

12

## Data Availability

Data sharing is not applicable to this article as no new data were created or analyzed in this study.
